# Mauriac Syndrome: A Rare Condition With Cushingoid Feature and Hepatomegaly in Poorly Controlled Diabetes Mellitus Type 1

**DOI:** 10.7759/cureus.65057

**Published:** 2024-07-21

**Authors:** Krutik J Brahmbhatt, Meet J Doshi, Milauni Dave, Het Contractor, Kush Shah

**Affiliations:** 1 Medicine, Government Medical College, Baroda, Vadodara, IND

**Keywords:** hepatomegaly, insulin treatment, hyperglycaemia, hepatic glycogenosis, diabetes type 1

## Abstract

Hepatic glycogenosis (HG) is a rare complication of poorly controlled type 1 diabetes mellitus (T1DM), in which glycogen accumulates in the hepatocytes. It can be caused by excessive insulin doses or recurrent ketoacidosis episodes. Mauriac’s syndrome is a rare disease that includes short stature, growth maturation delay, dyslipidemia, moon facies, protuberant abdomen, and hepatomegaly with transaminase elevation. It is clinically classified into two varieties based on the presence or absence of obesity and cushingoid appearance. Clinical, laboratory, and histological abnormalities are reversible with appropriate glycemic control. Our case is a 17-year-old male who had been a known case of T1DM for 15 years and presented with complaints of blurring of vision, facial puffiness, frequent urination, breathlessness, and generalized abdominal pain. Patient examination revealed cushingoid facies, abdominal distension due to hepatomegaly, and stunted growth with an altered lipid profile. He showed a very high sugar reading and was admitted for diabetic ketoacidosis. He was explained the proper diet and insulin administration technique and discharged with proper insulin dosages. On management, he showed normalization of liver enzymes and improved fat distribution with normal liver size. Thus, Mauriac syndrome is a reversible glycogen storage disease that can be completely managed with strict and continuous glycemic control in prepubertal T1DM patients.

## Introduction

Type 1 diabetes mellitus (T1DM) is an autoimmune condition commonly affecting people of young age. It typically causes hyperglycemia due to the pancreatic insufficiency to produce insulin. It is usually managed by exogenous insulin injections. Despite advancements in insulin therapy, poorly controlled T1DM can still lead to rare complications such as Mauriac syndrome, highlighting the need for vigilance in management. It is named after the scientist Mauriac, who defined the condition in 1930 [1].

Mauriac syndrome is typically specified by cushingoid appearance, short stature, growth and pubertal delay, and hepatomegaly, along with abnormally elevated liver enzymes and lipid profiles [2]. Hepatomegaly is the most striking feature of Mauriac syndrome, which is caused by an abnormal excess accumulation of glycogen in the liver and can be diagnosed with a liver biopsy or with minimally invasive gradient-dual-echo magnetic resonance imaging (MRI) [3]. It has been noted that with a continuous insulin delivery regime, there is a complete clinical, laboratorial, and histological remission [4]. The authors hereby present a case of a 17-year-old with Mauriac syndrome.

## Case presentation

A 17-year-old male presented to the outpatient department of our hospital. He had been a known case of T1DM for 15 years. The patient presented with chief complaints of blurring of vision for 1.5 years, facial puffiness for eight days, frequent urination for eight days, breathlessness for seven days, and generalized abdominal pain since the morning of the day of presentation. No complaints of fever, headache, vomiting, chest pain, or cola-colored urine. He was currently on a bolus basal regimen of insulin with 10U before breakfast and 10U before dinner, along with 15U glargine injections every alternate day.

**Figure 1:**
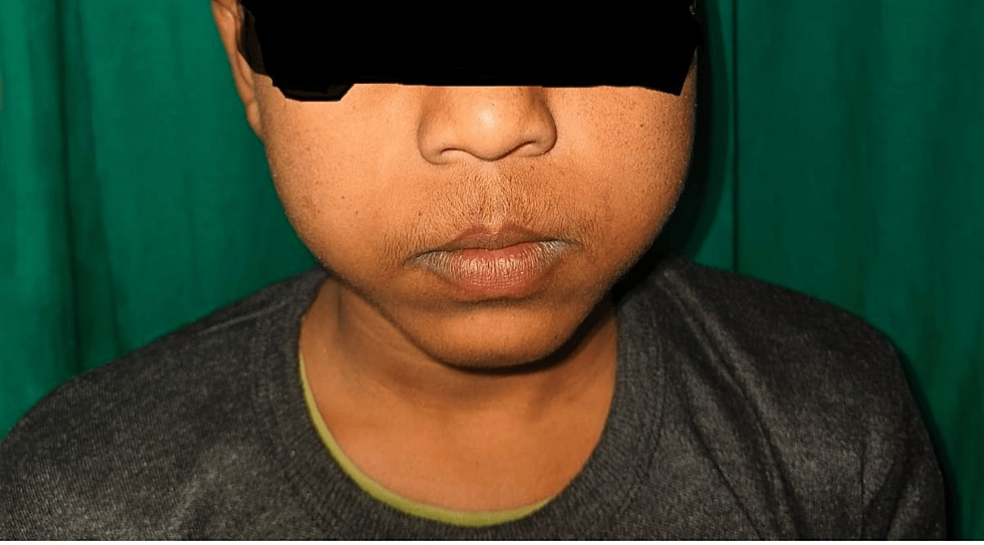
Cushingoid appearance of the face can be seen. Moon facies develop due to elevated corticosteroid levels.

On admission, he was conscious and well-oriented to time, place, and person. Vitals were as follows: pulse rate of 112 beats/min, blood pressure of 110/76 mm Hg, and respiratory rate of 18/min. Physical examination of the patient revealed a cushingoid appearance of the face as seen in Figure 1, stunting, and pubertal delay (height: 130 cm (<third percentile), weight: 30 kg (<third percentile), BMI: 14.7). Tanner staging revealed pubic hair stage 1 and testicles stage 1 (bilateral (B/L) volume < 4 ml). Random blood sugar sampling revealed 525 mg % of glucose. Abdominal examination revealed a clinically palpable enlarged liver 5 cm below the costal margin with no spleen enlargement or free abdominal fluid.

**Figure 2:**
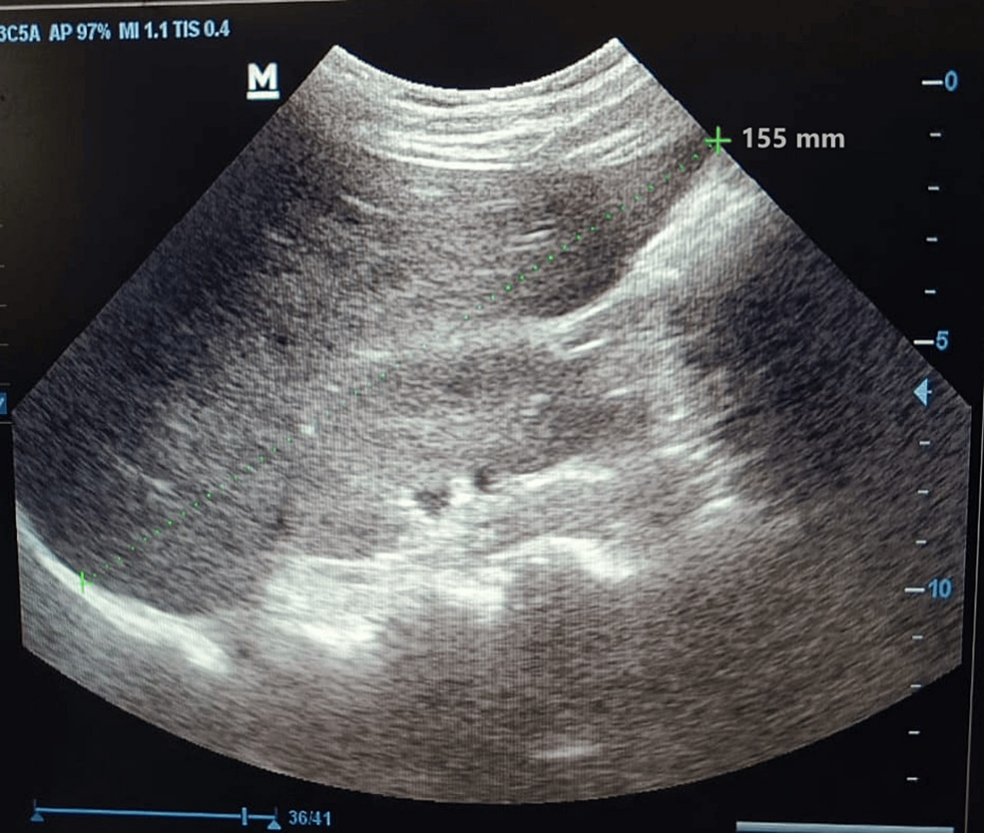
The image shows ultrasound of liver with a liver span of 155 mm indicating hepatomegaly. The dotted line (in green) represents the liver span equal to 155 mm.

Routine blood investigations revealed microcytic hypochromic anemia with low serum globulin levels (2.28 mg/dL). His glycosylated hemoglobin A1 (HbA1c) level was 15%. Arterial blood gas examination revealed partially compensated anion gap metabolic acidosis with partial pressure of carbon dioxide (pCO_2_) of 28.2 mm Hg and cHCO_3_(P) of 11.7 mmol/L. His hepatic panel revealed raised levels of alanine transaminase (138 UI/L), aspartate transaminase (124 UI/L), and alkaline phosphatase (176 UI/L). Urine examination reveals positive urine glucose 3+ and ketones 2+, microalbuminuria of 581 mg/day, creatinine concentration of 49.5 mg/dL, and a microalbuminuria:creatinine ratio of 1174.45 mg/gm. Hormonal investigation revealed slightly elevated serum cortisol levels (27.93 mcg/dL). Ultrasonography of the abdomen revealed an enlarged liver with no other abnormalities as seen in Figure 2. Investigations can be seen in Table 1.

**Table 1:** Laboratory and radiological investigations. U: units, IU: international units, +: mild, ++: moderate, +++: high, - : subjective reference for each parameter, ALT: alanine transaminase, AST: aspartate transaminase, ALP: alkaline phosphatases, FSH: follicle-stimulating hormone, LH: luteinizing hormone, HbA1c: glycosylated hemoglobin A1, pCO_2_: partial pressure of carbon dioxide.

Laboratory Analysis	Results	Reference Range
Peripheral smear	Microcytic hypochromic anemia	Volume <80fl
Hemoglobin	10.78 mg/dL	13-18 mg/dL
HbA1c	15 gm%	4.5-6.2 gm%
pCO_2_	28.2 mm Hg	35-45 mm Hg
CHCO^3-^(p)	11.7 mEq/L	22-28 mEq/L
ALT	138 U/L	10-40 U/L
AST	124 U/L	12-38 U/L
ALP	176 U/L	25-100 U/L
Urinalysis	Protein+, Ketones++, Glucose+++	-
Microalbuminuria	581 mg/day	<150 mg/day
Creatinine	49.5 mg/dL	20-200 mg/dL
Microalbuminuria:creatinine ratio:	1174.45 mg/gm	30-300 mg/gm
Serum cortisol	27.93 mcg/dL	5-25 mcg/dL
Serum FSH	5 IU/L	4-25 IU/L
Serum LH	8 IU/L	6-23 IU/L
Serum testosterone	127 ng/dL	100-1200 ng/dL

On the basis of clinical history and evaluation supported by laboratory investigation, a definitive diagnosis of Mauriac syndrome was made. For management, continuous insulin delivery was planned to avoid retinopathic complications. The patient’s random blood sugar was monitored five times a day. The patient was started on glargine (10U) and increased to 26U gradually, given subcutaneously once a day at night along with rapid-acting insulin of 12U before breakfast, 18U before lunch, and 18U before dinner. Along with that, the patient was started on prophylactic I/V ceftriaxone (12.5 mg/kg) twice a day and 1 ampule optineuron twice a day. The patient was kept indoors for seven days, during which he developed glycemic control, and his liver shrank back gradually to its physiological size.

## Discussion

Mauriac syndrome was a well-known condition in the 1900s, most commonly seen in patients with poorly controlled juvenile diabetes mellitus with evidence of multiple diabetes-related sequelae. It is rarely seen these days due to improved glycemic control with the help of improved varieties of long-acting insulin. It is also known as hepatic glycogenosis and is said to be caused by a mutation in PHKG2, which is the catalytic subunit of the enzyme glycogen phosphorylase kinase (PhK) [5]. PhK is a large enzyme complex responsible for the activation of glycogen phosphorylase. Because of this mutation, a significantly large amount of glycogen remains stored in the liver, hence the name hepatic glycogenosis [5]. The pathophysiology of Mauriac syndrome involves poor glycemic control leading to alternating hyperinsulinemia and hyperglycemia, resulting in glycogen accumulation in the liver and elevated corticosteroid levels contributing to a cushingoid appearance [2]. This causes elevated corticosteroid levels and is hence the most probable cause of cushingoid appearance, although the exact mechanism is still not clear [6]. Growth retardation is another typical finding in Mauriac syndrome, occurring because of the suppression of insulin-like growth factor 1 (IGF-1), leading to stunted growth. Mauriac syndrome remains underdiagnosed and often confused with non-alcoholic fatty liver disease (NAFLD), especially when Mauriac syndrome presents without a cushingoid appearance [7].

Mauriac syndrome is differentiated into two forms according to the presence or absence of obesity. The first form, which our patient had, is associated with the classical triad of dwarfism, obesity, and hepatomegaly. While the second form of Mauriac syndrome presents with hepatomegaly without obesity and persistent hyperglycemia [8], the second form is confused with NAFLD [1].

The goal of treatment lies in improving the metabolic status of the patient, which will automatically lead to improvements in related features of the syndrome like cushingoid features, growth, and pubertal delay. Theoretically and traditionally, intensified multiple insulin injection therapy is an approach that will cause rapid improvement in metabolic status but also greatly increase the risk of developing retinopathy [8].

Therefore, continuous insulin delivery resulting in a slow and step-by-step improvement of metabolic control is an acceptable method of improving metabolic status without increasing the risk of rapidly proliferative retinopathy. In our patient, we followed the regimen of continuous insulin therapy, which was given at doses of 12U, 18U, and 18U three times a day before meals, respectively, with continuous random blood sugar (RBS) monitoring.

## Conclusions

Mauriac syndrome in today’s era is an extremely rare complication developing due to repetitive hypo- and hyperinsulinemia over a long period of time. It should be highly suspected, especially in developing countries, due to poor affordability and resource-limiting conditions presenting with stunting, delayed puberty, and a history of recurrent diabetic ketoacidosis. Special attention should be given to rule out other differential diagnoses. The patient should be effectively managed with a continuous insulin delivery system, which reduces the variation of blood insulin levels, preventing diabetic ketoacidosis episodes as well as hyperinsulinemia.
